# Ammonia in Liver Disease: Diagnostic Utility, Preanalytical Challenges, Clinical Interpretation, and Therapeutic Implications: A Critical Narrative Review

**DOI:** 10.3390/diagnostics16213691

**Published:** 2026-11-09

**Authors:** Tamara Milovanovic, Anja Tomovic, Nikola Bakovic, Ivana Pantic, Marko Vojnovic

**Affiliations:** 1 Clinic for Gastroenterology and Hepatology, University Clinical Centre of Serbia, 11000 Belgrade, Serbia; 2 Faculty of Medicine, University of Belgrade, 11000 Belgrade, Serbia

**Keywords:** ammonia, hepatic encephalopathy, cirrhosis, diagnostic biomarker, preanalytical phase, clinical interpretation, prognosis, hyperammonaemia

## Abstract

Ammonia is central to the pathogenesis of hepatic encephalopathy (HE), but blood ammonia is neither a stand-alone diagnostic test nor a reliable measure of HE grade. Its clinical value depends on the population, the question being asked, and strict control of preanalytical and analytical conditions. This critical narrative review evaluates ammonia as a diagnostic and prognostic biomarker in liver disease, with emphasis on standardised measurement, interpretation in altered mental status, selective monitoring, and the distinction between biochemical response and patient-important outcomes. Evidence was updated through 9 October 2026 and prioritised international guidelines, the 2026 International Society for Hepatic Encephalopathy and Nitrogen Metabolism consensus, randomised trials, prospective cohorts, systematic reviews, and verified trial registries. In cirrhosis, an elevated value is nonspecific and does not confirm HE, whereas a methodologically reliable normal value should prompt diagnostic reassessment. Ammonia may add prognostic information in stable and decompensated cirrhosis, but thresholds and risk models require population-specific validation and do not independently mandate treatment. In acute liver failure, arterial ammonia has a distinct role in assessing the risk of cerebral oedema. Fasting before planned testing, avoidance of fist clenching and prolonged tourniquet use, rapid chilled transport, prompt plasma separation, and timely analysis are essential for an interpretable result. Treatment should be guided primarily by clinical course, correction of precipitants, and prevention of recurrence rather than serial ammonia concentrations. Standardisation and clinically anchored diagnostic algorithms are necessary before ammonia-guided management can be adopted broadly.

## 1. Introduction

The diagnostic use of blood ammonia remains one of the most frequently misunderstood areas in hepatology. Ammonia is biologically central to hepatic encephalopathy (HE), yet a peripheral concentration cannot by itself establish the diagnosis, determine West Haven grade, or distinguish HE from other causes of altered mental status. The clinically relevant questions are therefore not whether ammonia participates in pathogenesis, but when a measured value changes diagnostic probability, whether it adds prognostic information beyond established clinical variables, and whether ammonia-directed interventions improve patient-important outcomes. Biological plausibility, observational association, diagnostic utility, and therapeutic efficacy are different levels of evidence and should not be conflated. This review applies that distinction to current EASL and ACG guidance and to the 2026 ISHEN consensus on ammonia measurement [1,2,3].

## 2. Review Methodology

This article is a critical narrative review designed to address diagnostic interpretation rather than to estimate a pooled treatment effect. PubMed, journal and professional society websites, and ClinicalTrials.gov were searched and selectively updated through 9 October 2026. Search terms included ammonia, hyperammonaemia, hepatic encephalopathy, cirrhosis, acute liver failure, acute-on-chronic liver failure, sarcopenia, biomarker, preanalytical, measurement, point-of-care testing, and ammonia-lowering therapy. Priority was given to current guidelines and consensus statements, randomised trials, prospective cohorts, systematic reviews, and primary mechanistic studies. Registered studies were included to describe the development pipeline and were not treated as evidence of efficacy. Because the review was narrative, no formal meta-analysis or risk-of-bias synthesis was undertaken. Claims were calibrated to study design, prespecified endpoints, and clinical population.

## 3. Historical Development of the Ammonia Hypothesis

The association between liver disease and neuropsychiatric disturbances was recognised long before the biochemical basis of hepatic encephalopathy (HE) was established [4,5]. A pivotal experimental contribution came from a study of portosystemic diversion: in 1893, Hahn and colleagues reported neurological deterioration in dogs with an Eck fistula after nitrogen-rich feeding, linking portosystemic shunting with nitrogen load [6].

Clinical investigations in the 1950s subsequently connected nitrogenous substrates and elevated blood ammonia with hepatic coma and helped establish portosystemic encephalopathy as a clinical syndrome [7,8,9,10,11]. Later hypotheses involving false neurotransmitters and interactions with other neurotoxins broadened the original ammonia-centred model [12,13]. Contemporary understanding recognises interactions among hyperammonaemia, systemic inflammation, portosystemic shunting, skeletal muscle function, and cerebral susceptibility [14,15]. This multifactorial framework helps explain why peripheral ammonia concentrations correlate imperfectly with clinical HE severity.

## 4. Interorgan Ammonia Metabolism

Biological fluids contain a pH-dependent equilibrium between NH_3_ and NH_4_^+^. At physiological pH, more than 98% of the total is present in the ionised form. NH_3_ crosses membranes more readily, whereas NH_4_^+^ is also transported through specific transporters and ion channels. The laboratory term ammonia denotes the total concentration measured by the specific analytical method rather than the unionised fraction alone [16,17].

The gastrointestinal tract is an important source of portal ammonia. Enterocyte glutaminase degrades glutamine, bacterial ureases hydrolyse urea, and the breakdown of proteins and other nitrogenous substrates generates additional ammonia. The contribution of the small intestine is substantial; ammonia production should not be attributed exclusively to the colon. Gastrointestinal bleeding acutely increases the availability of nitrogenous substrates and may precipitate HE [18].

The liver uses sequential detoxification systems. Periportal hepatocytes convert ammonia into urea through a high-capacity, lower-affinity system. Pericentral hepatocytes, which are rich in glutamine synthetase, incorporate residual ammonia into glutamine through a high-affinity, lower-capacity system. Urea is eliminated predominantly by the kidneys. Glutamine is a temporary nitrogen carrier: its degradation in other organs releases ammonia again, and glutamine synthesis is therefore not equivalent to final nitrogen elimination [19,20,21].

**Figure 1:**
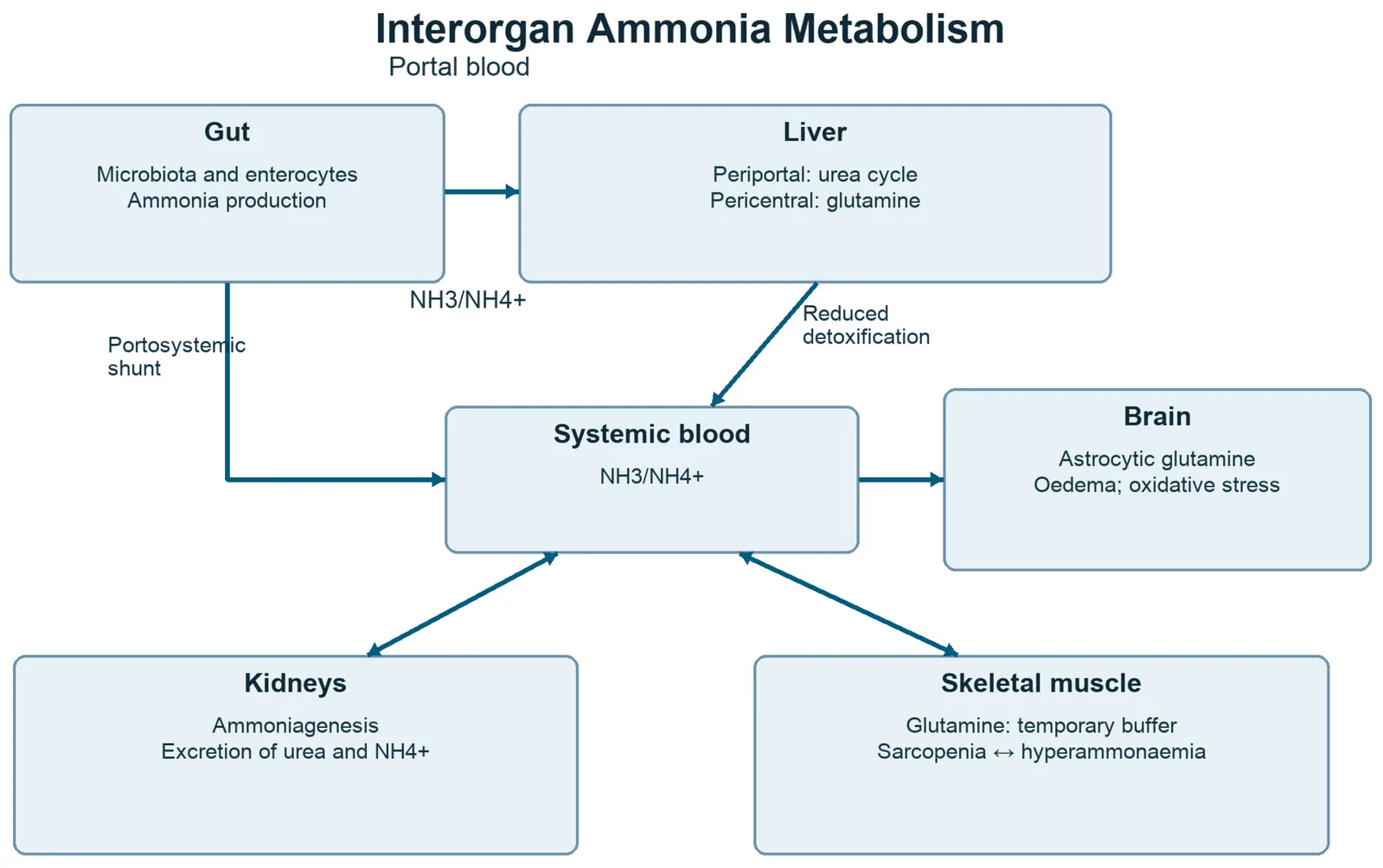
Interorgan ammonia metabolism and the consequences of portosystemic shunting. Glutamine synthesis temporarily sequesters nitrogen; final elimination occurs primarily through the excretion of urea and NH_4_^+^. Bidirectional arrows indicate nitrogen exchange. Original figure based on the cited sources [14,16,18].

The kidneys both generate ammonia from glutamine and excrete NH_4_^+^ in urine. The fraction excreted or returned to the systemic circulation depends on acid–base and potassium status. Hypokalaemia promotes renal ammoniagenesis, whereas renal dysfunction impairs overall nitrogen elimination. During hyperammonaemia, skeletal muscle assumes part of the detoxification burden through glutamine synthesis; this compensatory role depends on preserved muscle mass and function [16,18]. The main pathways of interorgan ammonia exchange are summarised in Figure 1.

## 5. Hyperammonaemia, Inflammation, and Sarcopenia

In cirrhosis, loss of functional hepatocyte mass and portosystemic shunting reduce hepatic clearance. Spontaneous shunts and transjugular intrahepatic portosystemic shunt (TIPS) placement may increase cerebral exposure without a proportional change in laboratory indices of hepatic synthetic function. Ammonia concentration therefore does not necessarily track with the MELD or Child–Pugh score. Infection, bleeding, constipation, dehydration, hypokalaemia, acute kidney injury, sedatives, and treatment nonadherence alter ammonia production, clearance, or cerebral susceptibility [1,14].

In astrocytes, glutamine formation and accumulation contribute to osmotic stress and swelling. The Trojan horse hypothesis describes mitochondrial uptake of glutamine and the subsequent release of ammonia, leading to oxidative and nitrosative stress and impaired bioenergetics. Altered GABAergic neurotransmission, neurosteroids, microglial activation, and dysfunction of the neuroglial vascular unit further shape the clinical phenotype. Systemic inflammation may amplify neurotoxicity at the same peripheral ammonia concentration [14,15].

Hyperammonaemia and sarcopenia form a bidirectional cycle. In experimental models, NF-κB activation and myostatin expression, inhibition of anabolic signalling, and increased autophagy disrupt muscle proteostasis. Muscle loss then reduces the reserve capacity for ammonia disposal. Ammonia lowering partly restores proteostasis, muscle mass, and strength in animal models, but these findings do not establish that every intervention with a biochemical effect improves function or survival in humans [22,23].

Emerging research has explored ammonia-related immune dysfunction and possible effects on fibrosis-associated pathways, although the supporting evidence remains predominantly preclinical or observational and does not establish ammonia as a clinical antifibrotic or anticancer target [15,24]. Multimarker approaches combining ammonia with inflammatory, muscle, and neuroglial variables are also under investigation and require prospective validation [25].

## 6. Diagnostic Interpretation Across Clinical Contexts

According to the underlying mechanism, HE is classified as type A in acute liver failure, type B due to portosystemic shunting without substantial parenchymal disease, and type C in cirrhosis. Covert HE comprises minimal HE and West Haven grade I, whereas overt HE comprises grades II–IV. Covert deficits in attention, psychomotor speed, and executive function may impair work, driving, and quality of life and increase the risk of falls. Diagnosis requires validated psychometric or neurophysiological tests adapted to language, age, and educational level [1,26].

Overt HE remains a clinical diagnosis based on compatible liver disease or portosystemic shunting, the time course, neurological examination, identification of precipitants, and exclusion of alternative causes. An elevated ammonia concentration is nonspecific: it neither confirms HE nor determines its West Haven grade. Conversely, a normal result obtained under reliable preanalytical conditions decreases the probability of HE and should trigger diagnostic reassessment, although it does not absolutely exclude the disorder. The result must therefore be interpreted as a probability-modifying biomarker rather than a binary diagnostic test [1,2,3]. The clinical meaning of an ammonia result in different settings is summarised in Table 1.

Minimal HE may occur with a normal ammonia concentration. Documentation of an episodic, recurrent, or persistent course and the presence of precipitating factors informs secondary prevention and assessment for transplantation. Disease burden also extends to caregivers, particularly in patients with repeated episodes and loss of independence [1,2].

**Figure 2:**
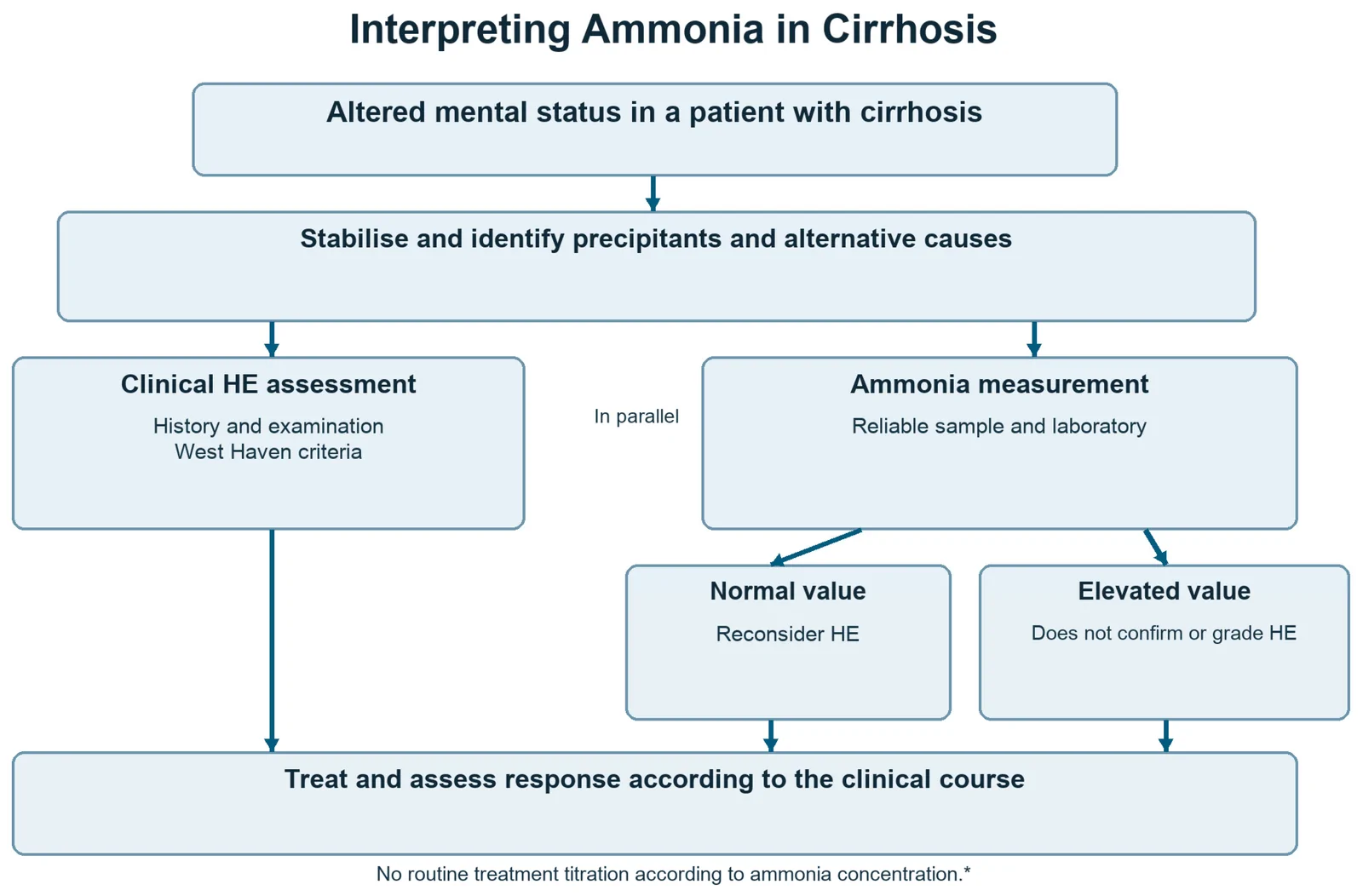
Clinical interpretation of ammonia in cirrhosis and altered mental status. A normal value does not absolutely exclude HE. * When response is inadequate, ISHEN recommends selective repeat measurement after 2–7 days together with renewed clinical assessment. Acute liver failure represents a separate clinical context. Original figure based on EASL, ACG, and ISHEN guidance [1,2,3].

The differential diagnosis includes sepsis, hypoglycaemia, sodium disorders, uraemia, hypercapnia, intoxication or withdrawal, adverse drug effects, Wernicke encephalopathy, nonconvulsive status epilepticus, and structural central nervous system lesions. Brain CT or MRI, electroencephalography, toxicology testing, and lumbar puncture should be selected according to the clinical phenotype and suspicion of an alternative diagnosis. Disproportionately severe impairment of consciousness, focal neurological deficits, seizures, or failure to respond as expectednecessitates broader evaluation [1,27]. A practical approach to the interpretation of ammonia in cirrhosis and altered mental status is shown in Figure 2.

**Table 1:** Clinical context determines the meaning of an ammonia result.

Context	What the Result may Provide	Limit of Inference
Suspected overt HE	A normal, reliable result should prompt reconsideration of the diagnosis.	An elevated result neither confirms HE nor determines West Haven grade.
Covert HE	Complementary information within the overall assessment.	Does not replace validated cognitive testing.
Stable cirrhosis	Population-level risk information and a possible component of a predictive model.	A threshold is not a stand-alone criterion for treatment or transplantation.
Inadequate response in hospitalised HE	Selective repeat testing after 2–7 days according to ISHEN 2026.	Does not constitute an algorithm for titrating lactulose or rifaximin.
Acute liver failure	Arterial ammonia contributes to assessment of cerebral oedema risk.	Thresholds from acute liver failure should not be transferred to cirrhosis.

HE, hepatic encephalopathy. Sources: [1,2,3,28].

## 7. Prognostic Utility and Limitations of Predictive Models

Prospective cohort studies in clinically stable cirrhosis have associated higher plasma ammonia with subsequent liver-related complications, hospitalisation, and mortality [29,30]. A focused review has synthesised the potential biomarker roles of ammonia in cirrhosis, while also highlighting the limitations of current thresholds and the distinction between diagnostic, prognostic, and treatment-response applications [31].

In acute decompensation and acute-on-chronic liver failure, ammonia has been associated with complications and mortality. These findings should be interpreted alongside the severity of organ dysfunction, infection, and disease aetiology. Thresholds and prognostic models developed in stable outpatients with cirrhosis cannot automatically be applied to critically ill populations [32].

AMMON-OHE combines ammonia expressed relative to the upper limit of normal with sex, diabetes, albumin, and creatinine. The original model was developed in 426 outpatients, and ammonia concentration was independently associated with the development of overt HE. Validation in additional cohorts supports its prognostic potential, but model discrimination does not establish that prophylaxis should be initiated in every patient without previous overt HE. The clinical utility of model-guided decisions requires direct testing, with appropriate citation of the original model and its subsequent validations [33,34].

Acute liver failure is a distinct setting: a rapid increase in arterial ammonia in the presence of limited osmotic adaptation is associated with cerebral oedema and intracranial hypertension. Values of approximately 150–200 µmol/L indicate increased risk in the appropriate clinical context, particularly with severe encephalopathy and renal dysfunction. These are not universal thresholds for cirrhosis or stand-alone criteria for transplantation [28,35]. 

## 8. Preanalytical Standardisation and Selective Monitoring

Preanalytical error can make an ammonia result clinically uninterpretable. Exercise, repeated fist clenching, prolonged tourniquet application, haemolysis, delayed plasma separation, and delayed analysis increase variability. Sample matrix, collection site, reference interval, and analytical platform should be documented, and results from different methods should not be compared uncritically. The 2026 ISHEN consensus recommendations are summarised in Table 2. Some procedural recommendations are supported by low-certainty evidence but strong expert agreement, reflecting the diagnostic harm caused by poorly controlled sampling [3,36].

Routine titration of treatment according to serial ammonia concentrations has not been validated. ISHEN nevertheless recommends repeat measurement after 2–7 days in hospitalised patients with overt HE who do not respond adequately to specific therapy, as an adjunct to reassessing the probability of resolution. This selective use differs from routine laboratory-guided management, which is not supported by the ACG [2,3].

Point-of-care devices may shorten processing time, but their use depends on analytical validation of the platform and sample matrix and on quality control. A study of a single analyser cannot be generalised to all devices, and analytical agreement does not demonstrate improved clinical outcomes [37].

## 9. Therapeutic Implications of Ammonia Measurement

In overt HE, priorities are patient stabilisation, airway protection when required, and treatment of precipitating factors. Lactulose is the cornerstone of therapy; the dose should be adjusted to clinical response and approximately two to three soft bowel movements per day. Excessive dosing that causes diarrhoea, dehydration, or electrolyte disturbances may worsen the clinical state. Lactulose is continued for secondary prevention after a first episode, and rifaximin is added when recurrence occurs despite maintained lactulose therapy [1,2,26].

The 2026 ACG guideline conditionally supports adding rifaximin to lactulose in acute overt HE. For elective TIPS, it recommends rifaximin beginning 14 days before the procedure and continuing for at least 6 months afterwards. This use should be distinguished from primary prevention in the broader population of patients with cirrhosis and no previous HE. The 2021 randomised trial by Bureau and colleagues provides direct evidence for prophylaxis in the specific TIPS population [2,38].

Polyethylene glycol with electrolytes may accelerate recovery in selected hospitalised patients; the ACG identifies it as an alternative to lactulose on the basis of a conditional recommendation and low-quality evidence. Aspiration risk, ileus, and haemodynamic status must be assessed. L-ornithine L-aspartate promotes ureagenesis and glutamine synthesis, but estimates of benefit are limited by heterogeneous routes of administration, HE phenotypes, and endpoints. Evidence that an intervention lowers ammonia should not be equated with evidence that it reduces mortality [2,39,40].

Ornithine phenylacetate enables urinary nitrogen elimination as phenylacetylglutamine. In a randomised phase 2b trial, it reduced ammonia but did not significantly improve the primary clinical endpoint in the overall analysed population. This illustrates the distinction between a pharmacodynamic effect and confirmed clinical efficacy. Strategies targeting the microbiota and bacterial urease remain investigational [41,42].

Ammonia-scavenging agents should be considered individually rather than as an interchangeable therapeutic class. With ornithine phenylacetate (OPA), phenylacetic acid combines with glutamine to form phenylacetylglutamine (PAGN), which is eliminated in urine. In a study of patients with acute liver injury or acute liver failure, urinary PAGN clearance was related to creatinine clearance, and conversion of phenylacetic acid to PAGN showed evidence of saturation as the OPA dose increased [43]. A population pharmacokinetic analysis likewise found that renal dysfunction reduced PAGN excretion, while hepatic function and body weight influenced phenylacetic acid exposure [44]. These findings make dose extrapolation in combined hepatic and renal dysfunction uncertain and argue against automatic dose escalation to achieve a laboratory target. They do not establish that OPA is uniformly ineffective in liver failure; rather, they demonstrate that its pharmacokinetics and nitrogen elimination depend on organ function. In cirrhotic patients with overt HE, the phase 2b randomised trial did not meet its primary clinical endpoint in the overall intention-to-treat population [41]. Accordingly, biochemical ammonia reduction should not be equated with proven clinical efficacy, and scavenger therapy should be selected and monitored according to the specific agent, clinical setting, and hepatic and renal function.

Nutritional and muscle-directed interventions are integral to management. Routine protein restriction should be avoided. For most patients, approximately 1.2–1.5 g protein/kg ideal body weight per day is recommended, together with individualised energy intake, several smaller meals, and a late-evening snack. Vegetable and dairy protein sources may be better tolerated. Physical activity should be adapted to functional status; a transient exercise-related increase in ammonia is not sufficient reason to avoid activity [2,45].

In refractory HE despite appropriate therapy, large spontaneous portosystemic shunts should be sought. Shunt embolisation is an option for carefully selected patients with sufficient hepatic reserve, with recognition of the risk of worsening portal hypertension. Advanced decompensated cirrhosis requires timely transplant assessment; the neuropsychiatric and functional burden of HE is not fully captured by the MELD score [1,27]. The main therapeutic strategies and their limitations are summarised in Table 3.

## 10. Recent Evidence Relevant to Diagnostic Interpretation

Recent studies should be interpreted according to the prespecified primary endpoint, sample size, and clinical population. A positive secondary or post hoc finding may justify further investigation but does not override a negative primary result. Table 4 summarises selected evidence relevant to ammonia and HE; the registered studies in the next section are protocols rather than evidence of efficacy.

Recent findings reinforce the need to distinguish biochemical, cognitive, and clinical outcomes. THEMATIC provides a promising signal for prevention of recurrence, subject to the limitations of a phase II study and post hoc analyses. In the small trial of rifaximin in covert HE, within-group improvement does not replace the primary comparison with the control group; the authors subsequently emphasised the limited statistical power [46,47,50].

A negative development result is also important. On 23 January 2026, the sponsor reported that both phase III RED-C trials of the rifaximin SSD formulation for prevention of a first HE episode did not meet their primary endpoint. Full peer-reviewed results were not presented in the sponsor communication. The finding pertains to the tested formulation and primary-prevention population and does not invalidate the established use of standard rifaximin with lactulose for prevention of recurrence [51].

## 11. Registered Studies and Development Programmes

Registered studies provide insight into research directions, but registration of a protocol does not constitute evidence of efficacy. Table 5 presents five selected registry entries and development programmes verified on 9 October 2026. The latest available update date is provided for each entry; formal registry status may lag behind the actual course of a trial. The selection is illustrative and is neither a complete inventory nor an estimate of the total number of active studies.

When quantifying research activity, the total number of registered studies should be distinguished from studies that are recruiting, enrolling by invitation, or active, not recruiting. Not yet recruiting studies represent planned research and should be reported separately. It should also be stated whether a count includes only interventional trials or also observational studies. Broader studies of cirrhosis in which HE is a secondary endpoint may be missed by a narrow search using Hepatic Encephalopathy as the condition.

PROMISE is an important research direction because it advances from small microbiota studies to a multicentre phase III trial. The protocol published in July 2026 describes a plan to enrol 300 participants with 24 months of follow-up. Its primary endpoint is infection or decompensation requiring an urgent assessment or hospitalisation; HE and mortality are neither the sole nor independent primary endpoints. The published protocol should therefore not be cited as a positive treatment result for HE [55].

In the RED-C programme, results were announced before the nominal registry status was aligned with the development outcome. This example shows why the number of studies actually in progress cannot be inferred from an outdated Active, not recruiting status alone. Update dates, published results, and sponsor communications must be considered together [51,57].

## 12. Discussion and Research Priorities

The current model links peripheral hyperammonaemia to a dynamic interorgan network in which inflammation, renal function, electrolytes, and muscle mass modify cerebral susceptibility. A single measured concentration captures neither the duration of exposure nor the intensity of the cerebral response. This explains how strong biological plausibility can coexist with limited specificity of the clinical test.

Prognostic association should be distinguished from clinical utility. A predictive model may discriminate risk groups effectively even though it has not yet been shown that using the model improves outcomes. Future multicentre studies should standardise sampling, prespecify phenotypes, and assess incremental value beyond MELD, Child–Pugh score, previous HE, and functional status. External validation should be followed by testing the clinical utility of model-guided decisions [33,34].

Therapeutic trials should distinguish ammonia lowering from cognitive recovery, prevention of recurrence, hospitalisation, quality of life, and survival. Research priorities include microbiota function and the safety of novel preparations, preservation of muscle mass, selection of patients for shunt intervention, and validation of reliable point-of-care tests. Outcomes important to caregivers, adherence, treatment access, and overall disease burden should also be included.

## 13. Conclusions

Ammonia is a central neurotoxin and a clinically informative marker of interorgan dysfunction, but its concentration is not a stand-alone test for HE. Diagnostic value depends on the clinical context and the reliability of collection, transport, processing, and analysis. In cirrhosis, an elevated result does not confirm HE, whereas a reliable normal result should prompt reconsideration of the diagnosis. Standardised measurement may support risk stratification and selected reassessment when response to treatment is inadequate; routine titration of therapy to serial concentrations is not justified. Future studies should evaluate whether standardised ammonia testing and validated multimarker models improve clinical decisions and patient-important outcomes rather than biochemical endpoints alone.

## Figures and Tables

**Table 2:** Standardised ammonia measurement in cirrhosis.

Step	Recommended Procedure
Preparation	At least 4 h of fasting for planned standardised measurement.
Sampling	Venous blood; report the sampling site. Plasma is preferred to serum or whole blood.
Preanalytics	Avoid prolonged tourniquet application, repeated fist clenching, and haemolysis.
Transport and processing	Transport on ice at approximately 4 °C; centrifuge and separate plasma within 15 min.
Analysis	Analyse as soon as possible and within 2 h; follow a validated laboratory procedure.
Reporting	Absolute value, unit, reference interval, and ratio to the upper limit of normal.

Source: ISHEN 2026 [3]. In acute altered mental status, preparation for planned measurement must not delay stabilisation or treatment.

**Table 3:** Therapeutic strategies and the clinical level of evidence.

Intervention	Place in Management	Main Limitation
Lactulose	Overt HE and secondary prevention.	Adjust dose to clinical response and bowel movements; avoid dehydration.
Rifaximin	Add-on therapy for recurrence; selected acute HE; prophylaxis with elective TIPS.	The indication is population-specific; results obtained with one formulation do not establish efficacy of another.
PEG with electrolytes	Selected hospitalised patients.	Small trials; caution regarding aspiration and fluid balance.
LOLA	Adjunctive therapy in an appropriate clinical context.	Heterogeneous evidence and insufficiently reliable effect on mortality.
Ornithine phenylacetate	Investigational nitrogen-scavenging therapy.	Negative primary endpoint in a phase 2b trial.
FMT	Investigational prevention of recurrence.	Efficacy and safety require confirmation in larger trials.
Nutrition and muscle function	Preservation of function and reserve nitrogen metabolism.	The optimal programme and direct effect on HE remain incompletely defined.
Shunt embolisation	Selected refractory HE.	Hepatic reserve and the risk of worsening portal hypertension limit use.

LOLA, L-ornithine L-aspartate; FMT, faecal microbiota transplantation. Sources: [1,2,27,39,40,41,45,46].

**Table 4:** Selected recent primary studies of ammonia and hepatic encephalopathy.

Study and Design	Main Result	Critical Interpretation
THEMATIC 2025 [46]. Double-blind phase II; N = 60; 6 months; FMT added to lactulose and rifaximin.	No FMT-related adverse events. HE recurrence: 9% with any FMT versus 40% with placebo; OR 0.15; 95% CI 0.04–0.64.	The primary endpoint was safety. Recurrence was a secondary endpoint, and the pooled comparison was post hoc. A larger confirmatory trial is required.
Inada et al. 2026 [47]. Open-label multicentre RCT; N = 50; covert HE; 12 weeks.	The primary between-group change in the Stroop test was not significant: −4.45 ± 7.12 versus −0.98 ± 5.74 s; *p* = 0.056. Composite events: 0/24 versus 7/26; *p* = 0.006. No significant change in ammonia.	Small unblinded sample and secondary composite endpoint. A positive article title does not alter interpretation of the primary endpoint.
Ling et al. 2026 [34]. Prospective cohort of stable cirrhosis; N = 280; 1 year.	Complications requiring hospitalisation: 60/280 (21.4%). Ammonia ≥ ULN: adjusted HR 4.92; 95% CI 2.80–8.64. AMMON-OHE AUROC 0.859; 95% CI 0.810–0.907.	The outcome was a composite of liver-related complications. The model underestimated absolute risk; discrimination and calibration should be assessed separately.
Jakhar et al. 2026 [42]. Translational study; 50 hospitalised patients without HE at baseline and 30 healthy controls; 3 months.	Overt HE developed in 20/50 patients. Faecal urease AUROC 0.78; normalised ammonia AUROC 0.71.	Small selected cohort without external validation. Statistical superiority of one AUROC and clinical benefit of urease inhibition in humans were not demonstrated.
Badal et al. 2024 [48]. Randomised study; N = 30 men; one meal containing 20 g protein; 3 h.	Ammonia increased after the meat meal, with smaller changes after vegetarian or vegan substitution. Time × diet interaction: *p* = 0.0009.	Short-term biochemical finding. There is no evidence of recurrence prevention, and the result does not justify restriction of total protein intake.
HEAL 2023 [49]. Double-blind RCT; N = 48; albumin versus saline; five weekly infusions.	Cognitive tests and psychosocial quality of life improved with albumin, without a significant change in ammonia or MELD.	Pilot study with a selected sample and short follow-up. Long-term prevention of overt HE and survival benefit were not established.

RCT, randomised controlled trial; N, number of participants; CI, confidence interval; OR, odds ratio; HR, hazard ratio; AUROC, area under the receiver operating characteristic curve; ULN, upper limit of normal; FMT, faecal microbiota transplantation.

**Table 5:** Selected registered protocols and development programmes relevant to hepatic encephalopathy.

Study and Sample	Intervention	Latest Reported Status	Primary Endpoint
HERB NCT06538077 Phase IV; planned N = 336 [52]	Oral BCAA versus rifaximin; both groups receive lactulose.	Recruiting, 10 June 2025.	Breakthrough overt HE over 24 weeks.
SUPPRESS-HE NCT07634237 Not applicable; planned N = 50 [53]	Personalised high-energy, high-protein diet after TIPS.	Not yet recruiting, 8 June 2026.	Incidence of overt HE over 6 months.
NCT06425380 Not applicable; planned N = 11 [54]	Resistant potato starch; open-label 4-week pilot.	Recruiting, 30 January 2026.	Change in faecal short-chain fatty acids; cognitive testing is a secondary endpoint.
PROMISE NCT06461208 Phase III; planned N = 300 [55,56]	FMT capsules versus placebo; multicentre, double-blind.	Recruiting according to ISRCTN, 13 February 2026. Protocol published 31 July 2026.	Infection or decompensation requiring urgent assessment or hospitalisation over 24 months. HE may be part of the composite; mortality is a secondary endpoint.
RED-C-3131 NCT05071716 Phase III; actual N = 524 [57]	Rifaximin SSD versus placebo; primary prevention.	Active, not recruiting, 24 July 2025. Negative result announced in 2026 [51].	First overt HE requiring medical intervention or death over 72 weeks.

BCAA, branched-chain amino acids; TIPS, transjugular intrahepatic portosystemic shunt; N, planned sample size, except for RED-C, for which actual enrolment is reported. PROMISE is a broader cirrhosis study, whereas RED-C illustrates the difference between formal registry status and an available development result.

## Data Availability

No new data were created or analysed in this study. Data sharing is not applicable to this article.

## References

[1] European Association for the Study of the Liver (2022). EASL Clinical Practice Guidelines on the management of hepatic encephalopathy. J. Hepatol..

[2] Bajaj J.S., Jakab S.S., Jesudian A.B., Rahimi R.S., Duarte-Rojo A., Chen P.-H., Wong R.J., Tapper E.B., Tandon P. (2026). ACG Clinical Guideline: Hepatic encephalopathy. Am. J. Gastroenterol..

[3] Ballester M.P., Gallego-Durán R., Shalimar, Thomsen K.L., Karvellas C.J., Dasarathy S., Shawcross D.L., Thabut D., Chen J., Rose C.F. (2026). Ammonia measurement in cirrhosis: International Delphi consensus recommendations from ISHEN. J. Hepatol..

[4] Amodio P. (2015). Hepatic encephalopathy: Historical remarks. J. Clin. Exp. Hepatol..

[5] Bass N.M. (2021). A brief history of hepatic encephalopathy. Clin. Liver Dis..

[6] Starzl T.E., Porter K.A., Francavilla A. (1983). The Eck fistula in animals and humans. Curr. Probl. Surg..

[7] Walshe J.M. (1951). Observations on the symptomatology and pathogenesis of hepatic coma. Q. J. Med..

[8] Phillips G.B., Schwartz R., Gabuzda G.J., Davidson C.S. (1952). The syndrome of impending hepatic coma in patients with cirrhosis of the liver given certain nitrogenous substances. N. Engl. J. Med..

[9] Sherlock S., Summerskill W.H.J., White L.P., Phear E.A. (1954). Portal-systemic encephalopathy; neurological complications of liver disease. Lancet.

[10] Phear E.A., Sherlock S., Summerskill W.H.J. (1955). Blood-ammonium levels in liver disease and hepatic coma. Lancet.

[11] Parsons-Smith B.G., Summerskill W.H.J., Dawson A.M., Sherlock S. (1957). The electroencephalograph in liver disease. Lancet.

[12] Fischer J.E., Baldessarini R.J. (1971). False neurotransmitters and hepatic failure. Lancet.

[13] Zieve L., Doizaki W.M., Zieve J. (1974). Synergism between mercaptans and ammonia or fatty acids in the production of coma. J. Lab. Clin. Med..

[14] Rose C.F., Amodio P., Bajaj J.S., Dhiman R.K., Montagnese S., Taylor-Robinson S.D., Vilstrup H., Jalan R. (2020). Hepatic encephalopathy: Novel insights into classification, pathophysiology and therapy. J. Hepatol..

[15] Gallego-Durán R., Hadjihambi A., Ampuero J., Rose C.F., Jalan R., Romero-Gómez M. (2024). Ammonia-induced stress response in liver disease progression and hepatic encephalopathy. Nat. Rev. Gastroenterol. Hepatol..

[16] Adeva M.M., Souto G., Blanco N., Donapetry C. (2012). Ammonium metabolism in humans. Metabolism.

[17] Sørensen M. (2013). Update on cerebral uptake of blood ammonia. Metab. Brain Dis..

[18] Levitt D.G., Levitt M.D. (2018). A model of blood-ammonia homeostasis based on a quantitative analysis of nitrogen metabolism in the multiple organs involved in the production, catabolism, and excretion of ammonia in humans. Clin. Exp. Gastroenterol..

[19] Häussinger D., Lamers W.H., Moorman A.F. (1992). Hepatocyte heterogeneity in the metabolism of amino acids and ammonia. Enzyme.

[20] Frieg B., Görg B., Gohlke H., Häussinger D. (2021). Glutamine synthetase as a central element in hepatic glutamine and ammonia metabolism: Novel aspects. Biol. Chem..

[21] Hakvoort T.B., He Y., Kulik W., Vermeulen J.L., Duijst S., Ruijter J.M., Runge J.H., Deutz N.E., Koehler S.E., Lamers W.H. (2017). Pivotal role of glutamine synthetase in ammonia detoxification. Hepatology.

[22] Qiu J., Thapaliya S., Runkana A., Yang Y., Tsien C., Mohan M.L., Narayanan A., Eghtesad B., Mozdziak P.E., McDonald C. (2013). Hyperammonemia in cirrhosis induces transcriptional regulation of myostatin by an NF-κB-mediated mechanism. Proc. Natl. Acad. Sci. USA.

[23] Kumar A., Davuluri G., e Silva R.N., Engelen M.P., Have G.A.T., Prayson R., Deutz N.E., Dasarathy S. (2017). Ammonia lowering reverses sarcopenia of cirrhosis by restoring skeletal muscle proteostasis. Hepatology.

[24] Kerbert A.J., Ballester M.P., Rose C.F., Jalan R. (2026). The role of ammonia in chronic liver disease. Hepatol. Commun..

[25] Gallego J.-J., Ballester M.-P., Fiorillo A., Casanova-Ferrer F., López-Gramaje A., Urios A., Arenas Y.M., Ríos M.-P., Durbán L., Megías J. (2025). Ammonia and beyond–biomarkers of hepatic encephalopathy. Metab. Brain Dis..

[26] Vilstrup H., Amodio P., Bajaj J., Cordoba J., Ferenci P., Mullen K.D., Weissenborn K., Wong P. (2014). Hepatic encephalopathy in chronic liver disease: 2014 Practice Guideline by the American Association for the Study of Liver Diseases and the European Association for the Study of the Liver. Hepatology.

[27] Kronsten V.T., Paintsil E.K., Rodrigues S., Seager M.J., Bernal W., Shawcross D.L. (2025). Hepatic encephalopathy: When lactulose and rifaximin are not working. Gastroenterology.

[28] Bernal W., Hall C., Karvellas C.J., Auzinger G., Sizer E., Wendon J. (2007). Arterial ammonia and clinical risk factors for encephalopathy and intracranial hypertension in acute liver failure. Hepatology.

[29] Tranah T.H., Ballester M.-P., Carbonell-Asins J.A., Ampuero J., Alexandrino G., Caracostea A., Sánchez-Torrijos Y., Thomsen K.L., Kerbert A.J., Capilla-Lozano M. (2022). Plasma ammonia levels predict hospitalisation with liver-related complications and mortality in clinically stable outpatients with cirrhosis. J. Hepatol..

[30] Shalimar, Sheikh M.F., Mookerjee R.P., Agarwal B., Acharya S.K., Jalan R. (2019). Prognostic role of ammonia in patients with cirrhosis. Hepatology.

[31] Ballester M.P., Durmazer E.N., Qi T., Jalan R. (2024). The value of ammonia as a biomarker in patients with cirrhosis. Semin. Liver Dis..

[32] Thanapirom K., Treeprasertsuk S., Choudhury A., Verma N., Dhiman R.K., Al Mahtab M., Devarbhavi H., Shukla A., Hamid S.S., Jafri W. (2024). Ammonia is associated with liver-related complications and predicts mortality in acute-on-chronic liver failure patients. Sci. Rep..

[33] Ballester M.P., Tranah T.H., Balcar L., Fiorillo A., Ampuero J., Kerbert A.J., Thomsen K.L., Escudero M.D., Mandorfer M., Reiberger T. (2023). Development and validation of the AMMON-OHE model to predict risk of overt hepatic encephalopathy occurrence in outpatients with cirrhosis. J. Hepatol..

[34] Ling L.P., Koh X.H., Zheng D.L.Y., Tan C.K., Wong Y.J., Lyn J.T.Y., Kwek A., Leong A.T., De Roza M., Keat T.H. (2026). Hyperammonemia predicts the development of liver-related events in a prospective cohort of stable cirrhosis patients and the external validation of the AMMON-OHE score. Hepatol. Commun..

[35] Duarte T., Fidalgo P., Karvellas C.J., Cardoso F.S. (2024). What every Intensivist should know about… Ammonia in liver failure. J. Crit. Care..

[36] Bowron A., Osgood V. (2024). Acceptability of plasma ammonia results when samples are not transported and processed under ideal conditions. Ann. Clin. Biochem..

[37] Rebollo-Calderón B., Calvo-López A., Ormazábal A., Artuch R., Rosell-Ferrer J., Alonso-Chamarro J., Puyol M. (2025). Evaluation of a point-of-care analyzer for blood ammonia determination. Anal. Bioanal. Chem..

[38] Bureau C., Thabut D., Jezequel C., Archambeaud I., D’aLteroche L., Dharancy S., Borentain P., Oberti F., Plessier A., De Ledinghen V. (2021). The use of rifaximin in the prevention of overt hepatic encephalopathy after transjugular intrahepatic portosystemic shunt: A randomized controlled trial. Ann. Intern. Med..

[39] Hoilat G.J., Ayas M.F., Hoilat J.N., Abu-Zaid A., Durer C., Durer S., Adhami T., John S. (2021). Polyethylene glycol versus lactulose in the treatment of hepatic encephalopathy: A systematic review and meta-analysis. BMJ Open Gastroenterol..

[40] He Q., Mao C., Chen Z., Zeng Y., Deng Y., Hu R. (2024). Efficacy of L-ornithine L-aspartate for minimal hepatic encephalopathy in patients with cirrhosis: A meta-analysis of randomized controlled trials. Arab J. Gastroenterol..

[41] Rahimi R.S., Safadi R., Thabut D., Bhamidimarri K.R., Pyrsopoulos N., Potthoff A., Bukofzer S., Bajaj J.S. (2021). Efficacy and safety of ornithine phenylacetate for treating overt hepatic encephalopathy in a randomized trial. Clin. Gastroenterol. Hepatol..

[42] Jakhar D., Shasthry S.M., Kumar G., Sharma A., Juneja P., Tripathi D.M., Sarin S.K., Kaur S. (2026). Gut microbial urease activity drives hyperammonemia and predicts overt hepatic encephalopathy in cirrhosis. J. Clin. Exp. Hepatol..

[43] Stravitz R.T., Gottfried M., Durkalski V., Fontana R.J., Hanje A.J., Koch D., Hameed B., Ganger D., Subramanian R.M., Bukofzer S. (2018). Safety, tolerability, and pharmacokinetics of L-ornithine phenylacetate in patients with acute liver injury/failure and hyperammonemia. Hepatology.

[44] Wang X., Vilchez R.A. (2022). Population pharmacokinetic analysis to assist dose selection of the L-ornithine salt of phenylacetic acid. Clin. Pharmacokinet..

[45] Lai J.C., Tandon P., Bernal W., Tapper E.B., Ekong U., Dasarathy S., Carey E.J. (2021). Malnutrition, frailty, and sarcopenia in patients with cirrhosis: 2021 Practice Guidance by the American Association for the Study of Liver Diseases. Hepatology.

[46] Bajaj J.S., Fagan A., Gavis E.A., Sterling R.K., Gallagher M.L., Lee H., Matherly S.C., Siddiqui M.S., Bartels A., Mousel T. (2025). Microbiota transplant for hepatic encephalopathy in cirrhosis: The THEMATIC trial. J. Hepatol..

[47] Inada H., Toyota T., Uojima H., Iio E., Miwa T., Miuma S., Miyase S., Mizuta T., Maeda D., Nagaoka K. (2026). Rifaximin improves cognitive performance and reduces cirrhosis-related adverse events in covert hepatic encephalopathy: A randomized controlled trial. Aliment. Pharmacol. Ther..

[48] Badal B.D., Fagan A., Tate V., Mousel T., Gallagher M.L., Puri P., Davis B., Miller J., Sikaroodi M., Gillevet P. (2024). Substitution of one meat-based meal with vegetarian and vegan alternatives generates lower ammonia and alters metabolites in cirrhosis: A randomized clinical trial. Clin. Transl. Gastroenterol..

[49] Fagan A., Gavis E.A., Gallagher M.L., Mousel T., Davis B., Puri P., Sterling R.K., Luketic V.A., Lee H., Matherly S.C. (2023). A double-blind randomized placebo-controlled trial of albumin in outpatients with hepatic encephalopathy: HEAL study. J. Hepatol..

[50] Inada H., Tanaka Y. (2026). Letter: Interpretation of cognitive and clinical outcomes in the randomized trial of rifaximin for covert hepatic encephalopathy. Authors’ reply. Aliment. Pharmacol. Ther..

[51] Bausch Health Companies Inc. (2026). Bausch Health Provides Update on RED-C Phase 3 Clinical Trials [Internet]. Sponsor Communication. https://ir.bauschhealth.com/news-releases/2026/01-23-2026-124516159.

[52] ClinicalTrials.gov (2025). Study Record NCT06538077. HERB [Internet]. NCT06538077.

[53] ClinicalTrials.gov (2026). Study Record NCT07634237. SUPPRESS-HE [Internet]. NCT07634237.

[54] ClinicalTrials.gov (2026). Study Record NCT06425380 [Internet]. NCT06425380.

[55] Kronsten V.T., Ntuli Y., Paintsil E.K., Woodhouse C.A., Tranah T.H., Cheung S., Hagan D., Merrick B., Cape A., Maher F. (2026). PROMISE: A PROspective randomised double-blind parallel group placebo-controlled multicentre trial of faecal MIcrobiota tranSplantation to improve outcomEs in patients with cirrhosis over 24 months—Study protocol for a clinical trial. Trials.

[56] ISRCTN (2026). ISRCTN17863382. PROMISE Trial [Internet]. ClinicalTrials.gov Identifier: NCT06461208. NCT06461208.

[57] ClinicalTrials.gov Study Record NCT05071716. RED-C-3131 [Internet]. Last Posted Update 24 July 2025; Results Submitted 29 September 2026. NCT05071716.

